# Influence of growth temperature on dielectric strength of Al_2_O_3_ thin films prepared via atomic layer deposition at low temperature

**DOI:** 10.1038/s41598-022-09054-7

**Published:** 2022-03-24

**Authors:** Suyeon Kim, Seung-Hun Lee, In Ho Jo, Jongsu Seo, Yeong-Eun Yoo, Jeong Hwan Kim

**Affiliations:** 1 Department of Materials Science and Engineering, Hanbat National University, Daejeon, 34158 Republic of Korea; 2 Department of Materials Science and Engineering, Korea Advanced Institute of Science and Technology (KAIST), Daejeon, 34141 Republic of Korea; 3 Department of Nano Manufacturing Technology, Korea Institute of Machinery and Materials (KIMM), Daejeon, 34103 Republic of Korea

**Keywords:** Materials science, Nanoscience and technology

## Abstract

Thin films grown via atomic layer deposition (ALD) suffer from insufficient growth rate and unreliability for temperature-sensitive electronic substrates. This study aimed to examine the growth characteristics and dielectric strength of ALD Al_2_O_3_ films grown at low temperatures (≤ 150 °C) for potential application in flexible electronic devices. The growth rate of the Al_2_O_3_ films increased from 0.9 to 1.1 Å/cycle with increasing temperature and saturated at growth temperatures ≥ 150 °C, which is the critical temperature at which a complete oxidation reaction occurred. The dielectric strength was also improved with increasing growth temperature, and the films grown at 150 °C showed a high breakdown field strength (~ 8.3 MV/cm), attributable to the decrease in the carbon impurities and oxygen defects, as confirmed by X-ray photoelectron spectroscopy. Even at low growth temperatures (≤ 150 °C), ALD Al_2_O_3_ films showed an overall amorphous structure and extremely smooth surfaces regardless of the growth temperature.

## Introduction

Aluminum oxide (Al_2_O_3_) films, as one of the most famous electronic materials, have been of interest in a variety of semiconductors, displays, sensors, and environment-friendly energy devices because of their high permittivity, excellent electrical insulation, wide bandgap, and superior encapsulation property^1–5^. Among the various growth techniques for fabricating Al_2_O_3_ films, atomic layer deposition (ALD), which is based on sequential and self-limiting reaction, has recently received much attention because of precise control over thickness and composition at the atomic level and exceptional conformality on 3-D structures even at low growth temperature^6,7^. With the emergence of flexible electronic devices, the demand for low-temperature deposition has rapidly increased, and ALD, which can be performed at a relatively low temperature, is expected to be extensively used for applications such as temperature-sensitive substrates that degrade above 200 °C^8^.

Although ALD has many advantages, there are still inherent issues, such as the insufficient growth rate and unreliability of the film, that need to be addressed before its deployment in low temperature fabrication processes. Unfortunately, the low productivity of ALD films is unavoidable because of their self-limiting behavior^9,10^. However, it is necessary to investigate and improve the electronic reliability, such as dielectric strength (which is the ability of an insulating material to withstand applied electrical stress) of the low-temperature-grown ALD films, which is very important for dielectric/insulating films in electronic devices.

To the best of our knowledge, a study on the dielectric strength of Al_2_O_3_ thin films prepared via ALD at low temperature has not been reported yet, despite its potential importance. Therefore, in this study, we experimentally investigated the growth features and dielectric strength of ALD Al_2_O_3_ films grown at low temperatures (≤ 150 °C), which are applicable to thermally unstable substrates. The growth characteristics were studied as a function of the temperature, which is a key growth parameter, and the ALD Al_2_O_3_ film properties were compared with those of ALD Al_2_O_3_ films grown at 250 °C, a sufficiently high temperature for the ALD Al_2_O_3_ formation reaction reported in previous publications^11,12^. In addition, the factors that can influence the dielectric strength were studied by examining the impurity level and structural defects in the films.

## Method

### Sample preparation

Al_2_O_3_ films were grown on a p-Si substrate and a sputtered Pt/Ti/SiO_2_/Si substrate using a traveling wave-type ALD reactor. The Pt/Ti/SiO_2_/Si substrate was sequentially cleaned with acetone, isopropanol, and deionized water prior to deposition. Meanwhile, the p-Si substrate was cleaned using a 10% diluted HF solution. Trimethylaluminum [TMA, Al(CH_3_)_3_], H_2_O, and Ar were used as the Al precursor, oxygen source, and purge gas, respectively. The Al_2_O_3_ film was grown in a substrate temperature range of 80–150 °C, and the control Al_2_O_3_ film was grown at 250 °C. The samples with Al_2_O_3_ films grown at 80, 100, 150, and 250 °C were designated as T80, T100, T150, and T250, respectively. All samples had film thicknesses of ~ 28 nm, controlled by the number of ALD cycles. The ALD process sequence was: Al[CH_3_]_3_ (1 s)–Ar purge (40 s)–H_2_O (2 s)–Ar purge (40 s).

### Characterization

The physical thickness and refractive index of the films were evaluated using a spectroscopic ellipsometer (MG-1000, Nano-View Co.). The film growth rate at each growth temperature was evaluated from the slope of the film thickness plot as a function of the number of ALD cycles. The film density and crystallinity were evaluated via X-ray reflectometry (XRR, NANOPIX, Rigaku) and X-ray diffraction (XRD, D/MAX 2500H, Rigaku) using a Cu Kα source. A scanning probe microscope (SPM, Nanoman , VEECO) was used to characterize the surface topography. Moreover, the impurity content in the films was analyzed using X-ray photoelectron spectroscopy (XPS, PHI 5000 VersaProbe, Ulvac-PHI) after removing the surface contaminants by Ar ion sputtering. The O 1s and C 1s core-level spectra were obtained to investigate the chemical state of the film. The atomic percentage of carbon impurity in the films was evaluated by dividing the area under the curve by the relative sensitivity factor of the C 1s peak from the XPS spectra and normalizing the value over the total amount of the film components.

Metal–insulator–metal (MIM) capacitors were then fabricated with an 80 nm-thick Au top electrode deposited through a shadow mask using a DC sputtering process to evaluate the electrical properties of the Al_2_O_3_ films. Dielectric constants of the films were evaluated using the capacitance measured by an impedance analyzer at a frequency of 100 kHz (HP4284A, Hewlett-Packard). A short-time test was conducted using a semiconductor parameter analyzer (4145B, Hewlett-Packard) to determine the breakdown voltage at a voltage ramp rate of 1.0 V/s.

## Results and discussion

**Figure 1:**
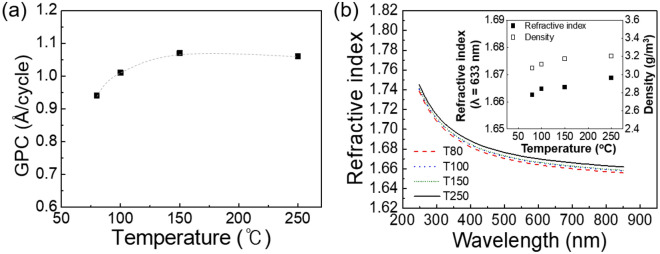
(**a**) Film growth per cycle as a function of growth temperature, (**b**) refractive index of the films as a function of wavelength measured using a spectroscopic ellipsometer. The inset shows the variation in the refractive index (measured at 633 nm) and film density (measured by XRR) with respect to the growth temperature.

Figure 1a shows the variation in the growth rate of ALD Al_2_O_3_ film with respect to temperature. The TMA and H_2_O purge time were fixed at 40 s. The growth rate of the Al_2_O_3_ film increased from ~ 0.9 Å/cycle at 80 °C to ~ 1.1 Å/cycle above 150 °C. Thus, the ALD window, which is the temperature range where the growth rate is constant, was established to provide a growth rate of ~ 1.1 Å/cycle at growth temperatures above 150 °C. Below 150 °C, the decrease in the growth rate with decreasing growth temperature could be attributed to the slower reaction rate because of the thermal activation barrier for the ligand (CH_3_) removal with liberating gaseous by-products^13^. Figure 1b shows the refractive indices of T80, T100, T150, and T250 measured in the wavelength range of 250–850 nm via spectroscopic ellipsometry. The indices increased slightly with increasing growth temperature and were similar to those reported in the literature^14–16^. The inset of Fig. 1b shows the film density and refractive indices at the wavelength of 633 nm as a function of the growth temperature. Refractive index values were lower than that of α-Al_2_O_3_ (sapphire, n = 1.766) at 633 nm. This is because the as-deposited Al_2_O_3_ thin films are amorphous and less dense than sapphire. All samples were amorphous, as confirmed by XRD and SPM analyses. Groner et al. reported that the refractive index and density of thin films are proportional through the Lorentz–Lorenz relationship^9^. Therefore, the density of the ALD Al_2_O_3_ films is expected to decrease slightly when the growth temperature decreases. In other reports, the density of the film grown via ALD largely decreases with decreasing growth temperature due to severely increasing content of residual impurities in the film^14,17^. However, only a slight reduction in the refractive index of the film with decreasing temperature was observed, indicating a slight decrease in the density, which is confirmed by the XRR results plotted in the inset of Fig. 1b. This small change in film density may have been caused by a small amount (< 2 at.%) of residual carbon impurity in the film grown at 80 °C, which can be proved by XPS analysis. This result also indicates that H_2_O is an appropriate source of oxygen for low-temperature ALD processes.

**Figure 2:**
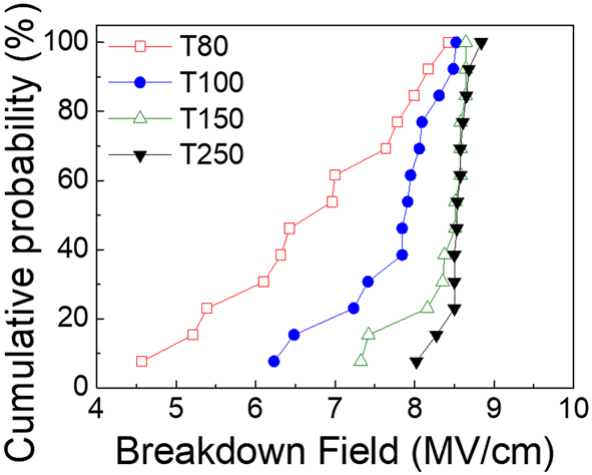
Cumulative probability of the electrical breakdown of ALD Al_2_O_3_ films grown at 80, 100, 150, and 250 ℃.

Figure 2 shows the cumulative probability of electrical breakdown of the Al_2_O_3_ films grown at various temperatures. A commonly used short-time test was conducted to evaluate the dielectric breakdown strength. The breakdown field was calculated by dividing the breakdown voltage by the thickness of each film. Meanwhile, the breakdown voltage is determined when the insulating property is severely degraded. In addition, it is noteworthy that ALD Al_2_O_3_ films for electrical characterization in this work were grown on Pt (~ 150 nm)/Si rather than on a Si substrate to avoid the complications originating from interfacial layer growth during deposition. As the growth temperature increased, the average breakdown field also increased, indicating a higher dielectric strength and a better quality insulator. Notably, T150 exhibited an improved average breakdown field of ~ 8.3 MV/cm, which was close to that of T250. The leakage current density characteristics of all the samples are shown in Figure S1. Furthermore, Table 1 shows the calculated standard deviation of the breakdown field of ALD Al_2_O_3_ films deposited at different growth temperatures. A decrease in the distribution of the breakdown field was observed as the growth temperature increased. A significant reduction in the distribution of the breakdown field was also confirmed for T150, compared to those for T80 and T100. The dielectric constants ranged from 7.1 to 7.6 across all temperature values, which are reasonable values compared to those of other reports^9,10^.

**Table 1:** Average breakdown field (E_BD_) strength and dielectric constant of ALD Al_2_O_3_ films grown at 80, 100, 150, and 250 ℃.

	T80	T100	T150	T250
Average E_BD_ (MV/cm)	6.8	7.7	8.3	8.5
Standard deviation	1.2	0.7	0.3	0.2
Dielectric constant	7.1	7.3	7.5	7.6

To determine the reason for higher dielectric strength with higher growth temperature, the residual carbon impurity level and chemical structure of the films were examined via XPS. Somewhat thick films (~ 28 nm) on Si enabled to obtain the signal from the bulk film, excluding that from the interface. The carbon impurity concentrations of T80, T100, T150, and T250 were 1.8, 1.3, 1.0, and 0.8, respectively (Table 2). The insufficient energy for ligand removal during ALD at low growth temperatures could contribute to the increased carbon content in the film, resulting in a slight decrease in the film density, as mentioned earlier (Fig. 1b). It has been reported that even in small amounts, carbon residue can degrade the reliability of ALD oxide films^18^. Therefore, the improved dielectric strength could be attributed to the decreased amount of carbon impurities in the films.

**Table 2:** Carbon concentration in the ALD Al_2_O_3_ films grown at 80, 100, 150, and 250 ℃.

	T80	T100	T150	T250
C (atom%)	1.8	1.3	1.0	0.8

**Figure 3:**
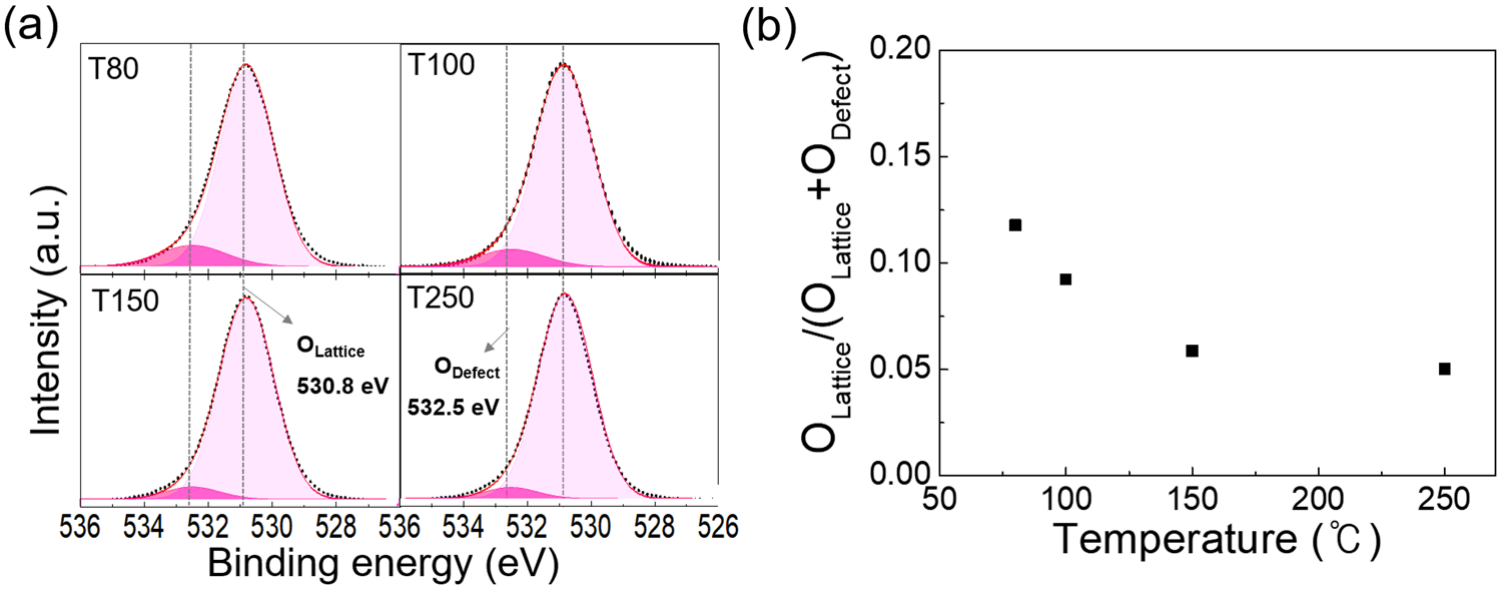
(**a**) O 1s XPS spectra and (**b**) the relative peak intensity [O_Defect_/O_Lattice_ + O_Defect_] of the O 1s XPS spectra of the ALD Al_2_O_3_ films grown at 80, 100, 150, and 250 ℃.

Figure 3a shows the O 1s core-level XPS spectra of the Al_2_O_3_ films grown at 80, 100, 150, and 250 °C. The O 1s peaks were deconvoluted into two sub-peaks corresponding to the lattice oxygen (O_Lattice_) and oxygen deficiency (O_Defect_) located at 530.8 and 532.5 eV^10,19^, respectively. As shown in Fig. 3b, the relative peak intensity [O_Defect_/O_Lattice_ + O_Defect_] abruptly decreased from 0.12 to 0.06 when the temperature increased from 80 to 150 °C, and slightly decreased from 0.06 to 0.05 when the temperature increased from 150 to 250 °C. It is known that the oxidation reaction is strongly influenced by thermal energy, depending on the deposition temperature. Therefore, the oxygen deficiency in the ALD film could be attributed to the incomplete oxidation in the ALD process due to insufficient thermal energy, which was more pronounced at low temperatures. In addition, oxygen defects, such as oxygen vacancies have been reported to form a conduction path and deteriorate the electrical properties, including the breakdown characteristics of the thin films^20,21^. Therefore, the improved dielectric strength at higher growth temperature, as shown in Fig. 2, could also be attributed to the reduction in oxygen deficiency in the films. Notably, a rapid decline in the relative peak intensities of the oxygen defect coincides with a significant reduction in the average and standard deviation values of the breakdown field below 150 °C. Consequently, more structural defects may be present in the Al_2_O_3_ film when grown at temperatures lower than 150 °C. Liu et al. reported that the presence of many structural defects in oxide films can lead to pore generation when the films are crystallized after high-temperature heat treatments^22^. This further confirms the potential pore generation in ALD Al_2_O_3_ films as the growth temperature decreases. Moreover, the presence of pores in the films may deteriorate the dielectric properties of ALD Al_2_O_3_ films.

**Figure 4:**
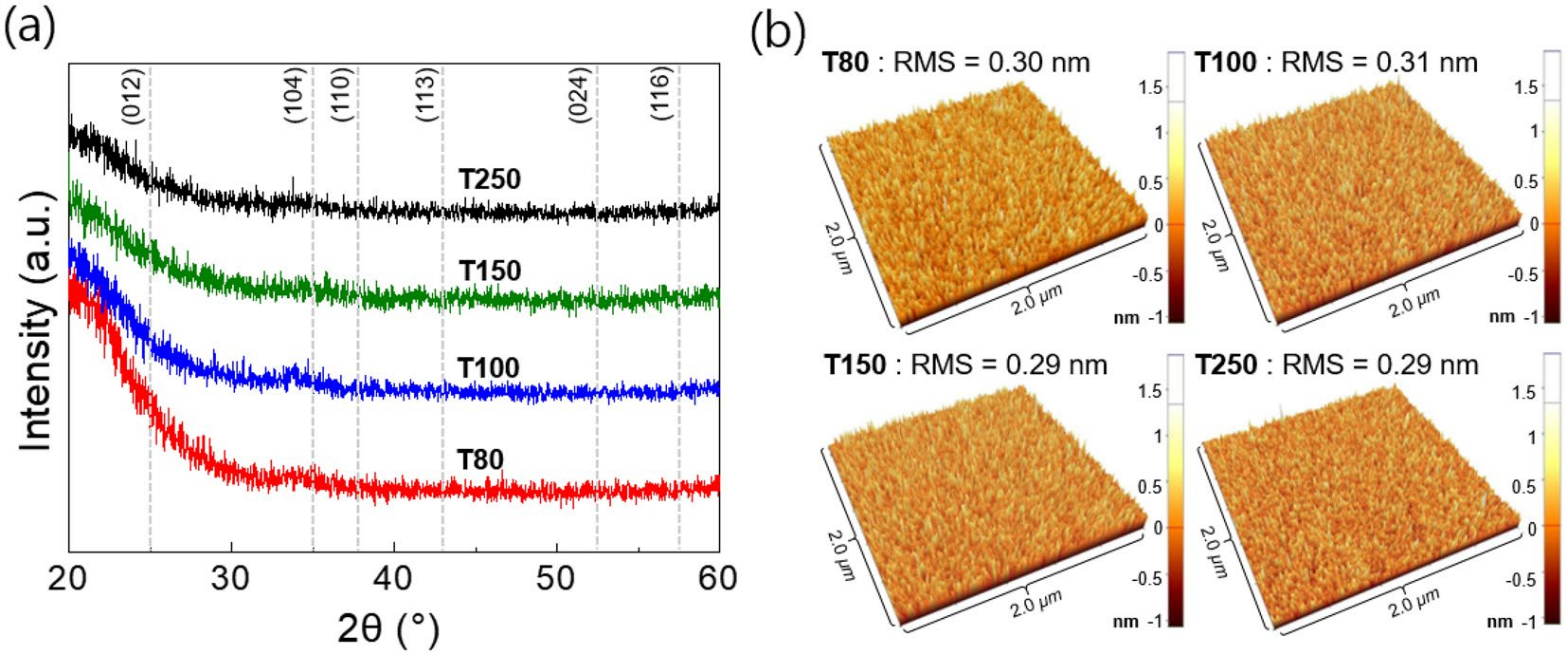
(**a**) XRD patterns and (**b**) SPM images with the RMS roughness of the Al_2_O_3_ films grown at 80, 100, 150, and 250 ℃.

It is worth examining whether the crystallinity and surface roughness can influence the dielectric strength of the films. XRD (in grazing angle incident mode) data in Fig. 4a show that all samples had an amorphous structure with no previously identified diffraction peaks, regardless of the growth temperature. Figure 4b shows the 2 μm × 2 μm SPM images of the Al_2_O_3_ films. The surfaces of all films were smooth without any sharp point that the electric field could focus on, which can influence the breakdown behavior. The films exhibited similar root mean square (RMS) roughness values (≤ 0.31 nm), which is similar to that of amorphous ALD Al_2_O_3_ films reported in a previous study^23^. Therefore, the influence of crystallinity and surface roughness on the dielectric strength could be considered negligible^9,19^.

## Conclusion

Al_2_O_3_ films were grown on a p-Si substrate and sputtered Pt/Ti/SiO_2_/Si substrate at 80, 100, 150, and 250 °C via ALD technique. An ALD window was established at deposition temperatures above 150 °C, which had a growth rate of 1.1 Å/cycle and a complete oxidation reaction with ligand removal. A slight increase in the refractive index of the film was observed with increasing temperature, indicating a slight increase in the film density, as confirmed by XRR, owing to the increased carbon impurities in the films. The higher amount of carbon impurities and oxygen defects also decreased the dielectric breakdown strength of the films below 150 °C. However, the films grown at 150 °C showed a breakdown field of ~ 8.3 MV/cm, which is suitable for flexible electronics because of the reduced carbon impurity level and oxygen defects in the films. The crystallinity and surface roughness of the ALD Al_2_O_3_ films remained unaffected by the growth temperature.

## Supplementary Information

Supplementary Information.
