# Supplementary material for: Influence of growth temperature on dielectric strength of Al2O3 thin films prepared via atomic layer deposition at low temperature
Source: Sci Rep. 2022 Mar 24;12:5124. doi: 10.1038/s41598-022-09054-7 (PMC8948174; doi:10.1038/s41598-022-09054-7)
Supplement: Supplementary file 1 — Supplementary Information. [file 41598_2022_9054_MOESM1_ESM.docx]

Supporting Information

Influence of growth temperature on dielectric strength of Al_2_O_3_ thin films prepared via atomic layer deposition at low temperature

*Suyeon Kim, Seung-Hun Lee, In Ho Jo, Jongsu Seo, Yeong-Eun Yoo, Jeong Hwan Kim*


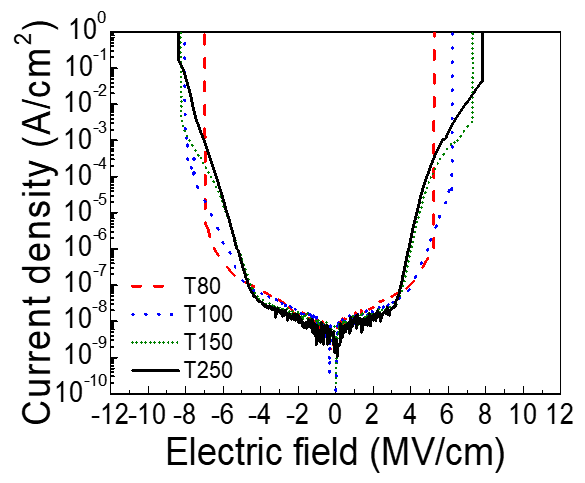


**Figure S1.** Leakage current density (*J-V*) of the ALD Al_2_O_3_ films grown at 80, 100, 150, and 250 °C


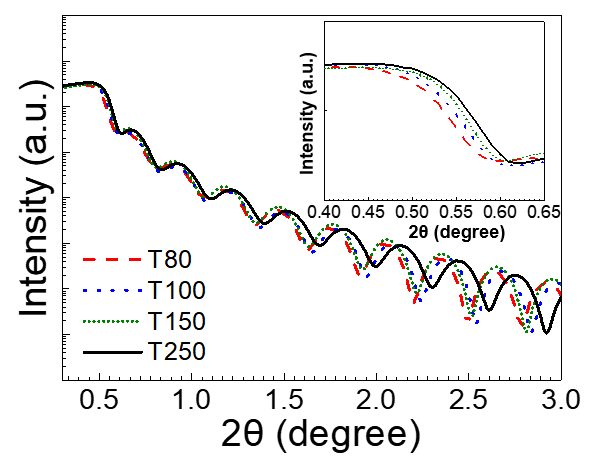


**Figure S2.** X-ray reflectivity (XRR) spectra of the ALD Al_2_O_3_ films grown at 80, 100, 150, and 250℃. The critical angles are shown in the inset.
